# Editorial: Mechanisms and novel therapeutic targets and approaches for mitochondrial dysfunction in neurological and cardiovascular diseases

**DOI:** 10.3389/fphys.2026.1840059

**Published:** 2026-04-21

**Authors:** Qiuxia Li, Quanjiang Zhang

**Affiliations:** Division of Endocrinology, Diabetes and Metabolism, Department of Medicine, David Geffen School of Medicine and UCLA Health, University of California, Los Angeles, Los Angeles, CA, United States

**Keywords:** cardiovascular disease, mitochondrial dysfunction, mitochondrial quality control, neurological disorders, therapeutic approaches

Healthy mitochondria are indispensable to eukaryotic cells, regulating numerous cellular processes, including energy and substrate metabolism, regulated cell death (RCD), and calcium homeostasis. Once dysfunctional, mitochondria impair cells by reducing ATP production, increasing reactive oxygen species (ROS) generation, and releasing mitochondrial contents to induce RCD. Mitochondrial dysfunction is a cause of various neurological and cardiovascular diseases. Consequently, cells evolve complex quality-control mechanisms to maintain mitochondrial health. These mechanisms involve mitophagy, fusion-fission dynamics, base excision repair (BER), and intercellular mitochondrial transfer. The articles in the Research Topic “Mechanisms and Novel Therapeutic Targets and Approaches for Mitochondrial Dysfunction in Neurological and Cardiovascular Diseases” delve into mitochondrial dysfunction in neurodegenerative and cardiovascular diseases and explore novel therapeutic avenues that specifically target mitochondrial dysfunction, with the ultimate goal of mitigating or halting the progression of these debilitating conditions.

Mutations in mitochondrial proteins are well-known mechanisms underlying mitochondrial dysfunction. Till now, 1136 proteins, of which 13 are encoded by mtDNA and the rest by nuclear DNA, have been identified in mitochondria (Human MitoCarta3.0; https://personal.broadinstitute.org/scalvo/MitoCarta3.0/human.mitocarta3.0.html). Numerous polymorphic variants in the genes encoding these proteins have been identified in patients with various diseases, including neurological and cardiovascular diseases, some of which are pathogenic in disease development by impairing mitochondria. By Dec 2025, MITOMAP has recorded 592 mtDNA variants in total, of which 16 are pathogenic, and 44 are likely pathogenic (https://www.mitomap.org/foswiki/bin/view/MITOMAP/MutationsCodingControl). ClinVar has recorded 300,614 variants in unclear DNA-encoding mitochondrial genes (NEMG), of which a small number are pathogenic, and most are of uncertain significance or likely benign. This record is still accumulating. In the Research Topic, Garre et al., by retrospective and cross-sectional analysis of European ethnicity in the UK Biobank, identified 7 mtDNA variants and 66 NEMG variants that are statistically associated with cardiovascular disease. Notably, 7 mtDNA variants have been recorded in ClinVar and classified as benign/likely benign. All NEMG variants have been recorded in the NIH dbSNP database and are intronic. In addition, recent studies have indicated the associations between some variants and cardiovascular disease, such as NOS3 (rs3918226, C>T) in myocardial infarction and hypertension (Al-Ali et al., 2023; Emdin et al., 2018). Although the various association analyses have been performed, the causal relationship between these variants and cardiovascular disease remains to be experimentally validated.

Mitochondrial function may be regulated by some non-mitochondrial proteins through their multiple targeting. One of these proteins is caveolins, an integral membrane protein. One of the well-known molecular functions of caveolins is to form caveolae, thereby regulating numerous cellular processes, including receptor-mediated signal transduction, macromolecular uptake, and cytoskeletal organization. Beyond that, studies have evidenced their distribution in organelles such as mitochondria and nucleus and functional significance in a caveolae-independent fashion (Fridolfsson et al., 2012; Goutas et al., 2021; Li et al., 2001; Lolo et al., 2023; Mundy et al., 2012). Although it is arguable whether all three caveolins are expressed in cardiomyocytes, their importance for cardiac health and disease has been well established. In the Research Topic, Zadorozyn et al. summarize the advances in pathophysiological roles of caveolins in cardiac health and the development of cardiac disease, including myocardial ischemia, heart failure, diabetes-induced metabolic cardiomyopathy, and septic cardiomyopathy. Beyond this review article, caveolins have been involved in the development of vascular and neurological diseases (Mathew, 2021; Wang et al., 2025). Given the current experimental strategies, which are mostly based on genetic knockout and overexpression, it is likely that the roles of caveolins in cardiovascular and neural health and diseases are attributable to the combination of caveolae-dependent and -independent functions. Because the mechanisms underlying the localization of caveolins to various organelles remain to be characterized, it is unlikely to separately characterize caveolins’ caveolae-dependent and -independent functions.

Some metabolic intermediates, such as aldehydes, are highly reactive and impair cells. Aldehydes, carbonyl compounds, including 4-HNE, MDA, and acrolein, may react with DNA, proteins, and lipids, etc., which have been implicated in the development of neurological and cardiovascular diseases. Aldehyde dehydrogenase 1 (ALDH1) detoxifies aldehydes by oxidizing them to carboxylic acids. In the Research Topic, Zhao et al. summarize the biological function of the ALDH1 family and its pathogenic role in related diseases, including Parkinson’s disease (PD) and cardiovascular disease. ALDH1 has six isoenzymes (ALDH1A1, ALDH1A2, ALDH1A3, ALDH1B1, ALDH1L1, and ALDH1L2), of which ALDH1B1 and ALDH1L2 are localized in mitochondria, while the others are in the cytosol. All ALDH1 isoenzymes are expressed in the brain, with varying levels across regions. ALDH1A2, ALDH1B1, ALDH1L1, and ALDH1L2 are detectable in the heart at varying levels, but ALDH1A1 and ALDH1A3 are undetectable (Human Protein Atlas). Beyond this review, studies have indicated that ALDH1A1 exacerbates neurodegeneration of dopaminergic (DA) neurons within the substantia nigra pars compacta (SNpc) in a PD mouse model (Liu et al., 2014) and that patients with ALDH1L2 mutation reveal neuro-ichthyotic syndrome (Sarret et al., 2019). However, mice deficient in ALDH1L2 do not display noticeable alterations in the brain and heart (Krupenko et al., 2020), but that loss of ALDH1A1, ALDH1A2, and ALDH1A3 increases cardiomyocyte apoptosis induced by infarction (Da Silva et al., 2021), implying that ALDH1A2 loss predisposes hearts to stress-induced injury. Although undetectable in cardiomyocytes, ALDH1A1 loss induces aortic valve calcification (Rosa et al., 2025). Of note, it remains unknown whether the mitochondrial quality control (MQC) system is involved in aldehyde-induced impairments.

Eukaryotic cells maintain mitochondrial health through MQC, and any mediators that regulate MQC may affect mitochondrial homeostasis. In the Research Topic, two reviews focus on the MQC system and its significance in cardiac and neurological diseases, respectively. One by Zhang et al. summarizes advances in the regulation of mitochondrial homeostasis by Krüppel-like factors (KLFs) via the MQC, focusing on four aspects: mitochondrial biogenesis, fusion-fission dynamics, mitophagy, and UPRmt. Another study by Chen et al. summarizes the significance of natural products in regulating mitochondrial homeostasis via the MQC, involving, beyond MQC, mitochondria-dependent apoptosis. Importantly, these two reviews provide insights into therapeutic approaches targeting MQC. In addition, Zhang et al. summarize another 3 MQC mechanisms: (1) Mitochondrial-derived vesicles (MDVs) to selectively deliver oxidized products to lysosomes for degradation (Neuspiel et al., 2008; Soubannier et al., 2012); (2) Mitochondria spheroid formation to fuse with a lysosome (Ding et al., 2012a, Ding et al., 2012b); and (3) Mitocytosis to eliminate damaged mitochondria into the surrounding environment (Jiao et al., 2021). These two reviews do not summarize whether KLFs and natural products target MQC to maintain mitochondrial homeostasis via these 3 MQC systems. Recent studies have developed approaches to target mitocytosis to treat brain tumors (Xiang et al., 2024).

The identification of active ingredients with the potential to treat/prevent diseases is a hot topic in biomedicine. Herbal medicine is a valuable source of medicinal ingredients, and enthusiasm for screening and characterizing its active ingredients is on the rise. Till now, many active ingredients have been identified, for example, Gypenoside XLIX, an important metabolite of *Gynostemma pentaphyllum* saponins, with potential to treat renal fibrosis, liver injury, and sepsis. In the Research Topic, Liu et al. characterize Gypenoside XLIX in preventing stroke-induced brain injury. This study not only expands the biological function of Gypenoside XLIX but also elucidates underlying mechanisms by outlining its interaction with PI3K, thereby activating PI3K/Akt/FOXO1 signaling transduction to regulate mitophagy. In addition, another article in the Research Topic, Zeng et al., summarizes the advances of pterostilbene, a dimethoxylated derivative of resveratrol, in regulating mitochondrial retrograde signaling that has been shown to regulate the identity and maturation of metabolic tissues (Walker et al., 2025) and neuronal function and disease (Cagin et al., 2015; Granat et al., 2020). In line with the article by Chen et al. in the Research Topic, active ingredients from herbal medicine are a valuable strategy for targeting the MQC system in the treatment of neurological and cardiovascular diseases. Notably, the mechanisms by which active ingredients target the MQC system remain incompletely understood; for example, co-crystal structures of these active ingredients with their targets are lacking, which hampers our understanding of these active ingredients and their clinical application.

In addition to pharmaceutical and genetic methods, physiotherapeutic approaches have been shown to regulate mitochondrial homeostasis, including transcranial magnetic stimulation (TMS). In the Research Topic, Wang et al. summarize the application of TMS to improve neural function and treat cognitive impairment in neurodegenerative diseases. The improvement of mitochondrial homeostasis is considered one of the underlying mechanisms, involving reduction in mitochondrial oxidative stress, maintenance of mitochondrial membrane integrity, and activation of the PI3K/Akt/mTOR signaling pathway. In addition, the current studies have shown that TMS may target the MQC system by altering mitophagy, mitochondrial dynamics (Ong and Tang, 2025; Song et al., 2025). Although the underlying mechanisms remain to be investigated, TMS is a valuable tool for treating mitochondrial dysfunction-related neurological disease.

In summary, articles in the Research Topic “Mechanisms and Novel Therapeutic Targets and Approaches for Mitochondrial Dysfunction in Neurological and Cardiovascular Diseases*”* summarize and advance our understanding of mitochondrial dysfunction in the development of neurological and cardiovascular diseases. More significantly, the articles summarize some valuable therapeutics to ameliorate mitochondrial dysfunction and their potential for the treatment of neurological and cardiovascular diseases. However, the mechanisms underlying mitochondrial dysfunction in the setting of neurological and cardiovascular diseases remain to be investigated, which may significantly hamper efforts to understand how the therapeutics improve mitochondrial function. The insufficient understanding of mitochondrial dysfunction currently retards the exploitation and application of therapeutics for the treatment and prevention of neurological and cardiovascular diseases via targeting mitochondrial dysfunction. Predictably, as our understanding of mitochondrial dysfunction advances more comprehensively and precisely, more therapeutics with greater effectiveness will be developed to treat and prevent neurological and cardiovascular diseases by ameliorating mitochondrial dysfunction.
